# Supplementary material for: Biodegradable and Biocompatible Thermoplastic Poly(Ester-Urethane)s Based on Poly(ε-Caprolactone) and Novel 1,3-Propanediol Bis(4-Isocyanatobenzoate) Diisocyanate: Synthesis and Characterization
Source: Polymers (Basel). 2022 Mar 23;14(7):1288. doi: 10.3390/polym14071288 (PMC9002640; doi:10.3390/polym14071288)
Supplement: Supplementary file 1 [file polymers-14-01288-s001.zip › polymers-1633997-supplementary.pdf]

Biodegradable thermoplastic poly(Ester-Urethane) Based on Poly(ε-caprolactone) and Novel 1,3-propanediol bis(4-isocyanatobenzoate) diisocyanate: Synthesis and characterization

Authors: Alejandra Rubio Hernández-Sampelayo[1,2], Rodrigo Navarro[1], Dulce María González-García[3,4], Luis García-Fernández[1,5], Rosana Ramírez[1,5], María Rosa Aguilar[1,5], Ángel Marcos-Fernández[1].

1- Institute of Polymer Science and Technology, Juan de la Cierva, 3, 28006, Madrid (Spain)
2- Universidad Nacional de Educación a Distancia (UNED), C/Bravo Murillo, 38, 28015 Madrid, Spain
3- Instituto Politécnico Nacional, ESIQIE, UPALM-Zacatenco, Col Lindavista, 07738, Mexico City (Mexico)
4- Universidad de Guanajuato, Dpto. de Química, Noria Alta s/n, 36050, Guanajuato, México
5- Biomedical Research Networking Center in the subject area of Bioengineering, Biomaterials and Nanomedicine (CIBER-BBN), Madrid, Spain

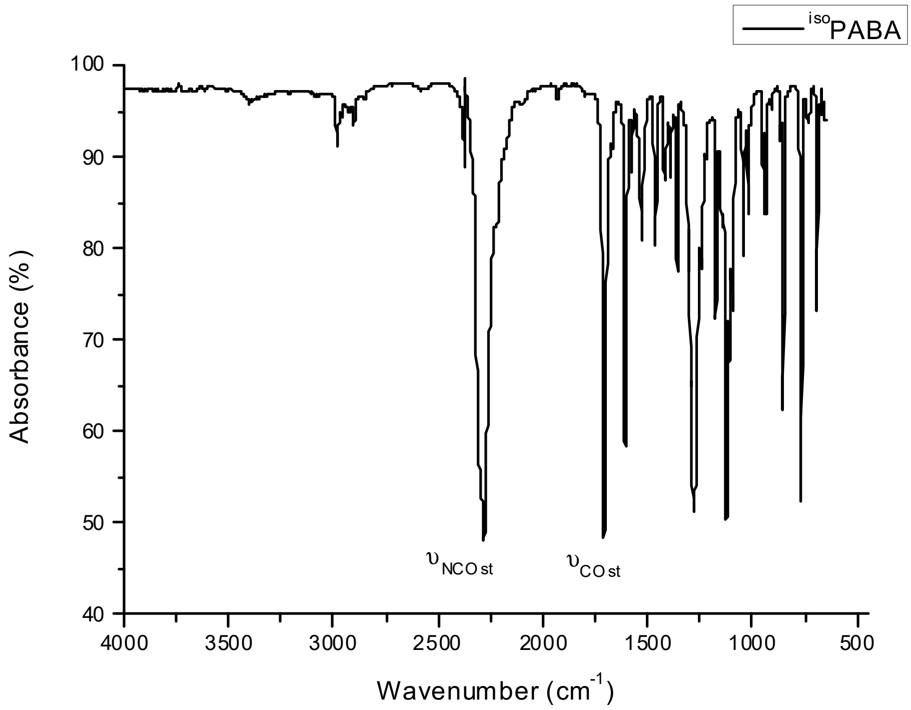

Figure S1: ATR-FTIR of aromatic diisocyanate isoPABA.

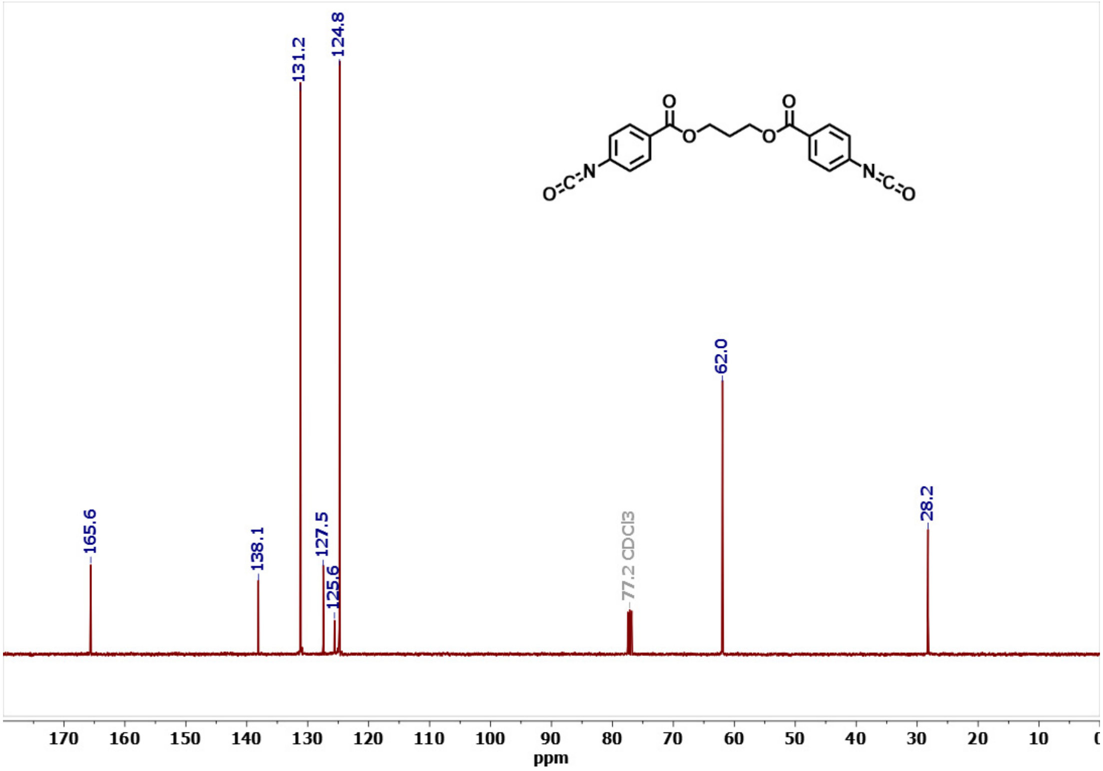

Figure S2: 13C-NMR spectrum of isoPABA in deuterated chloroform.

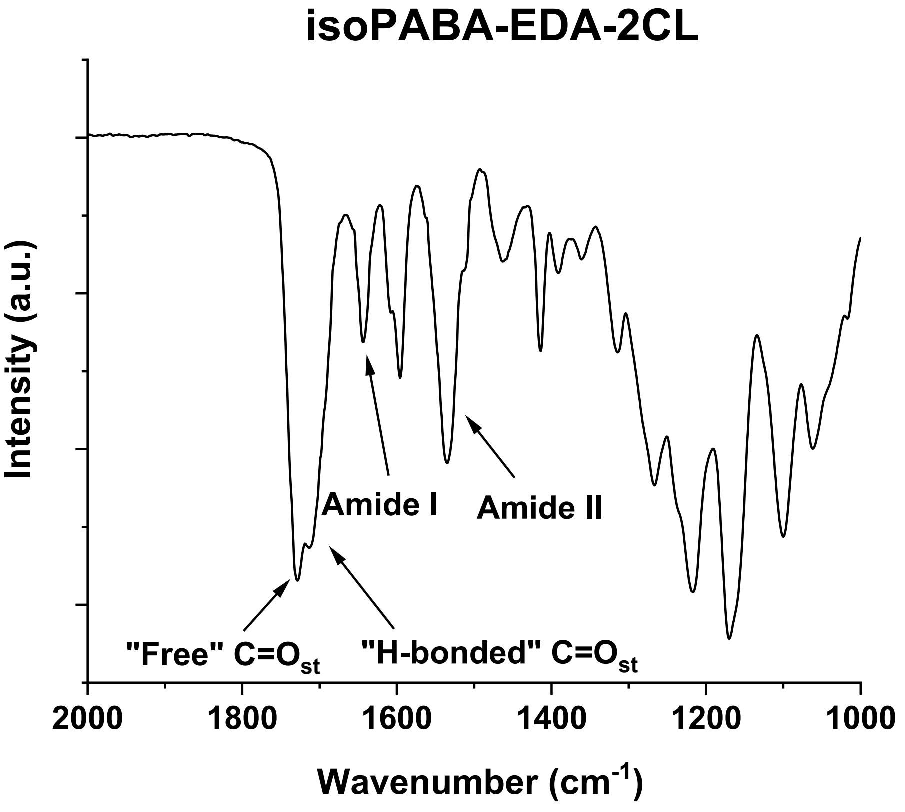

Figure S3. Extended FTIR-Spectrum between 2000 and 1000 cm-1 of model polyurethane isoPABA-EDA-2CL.

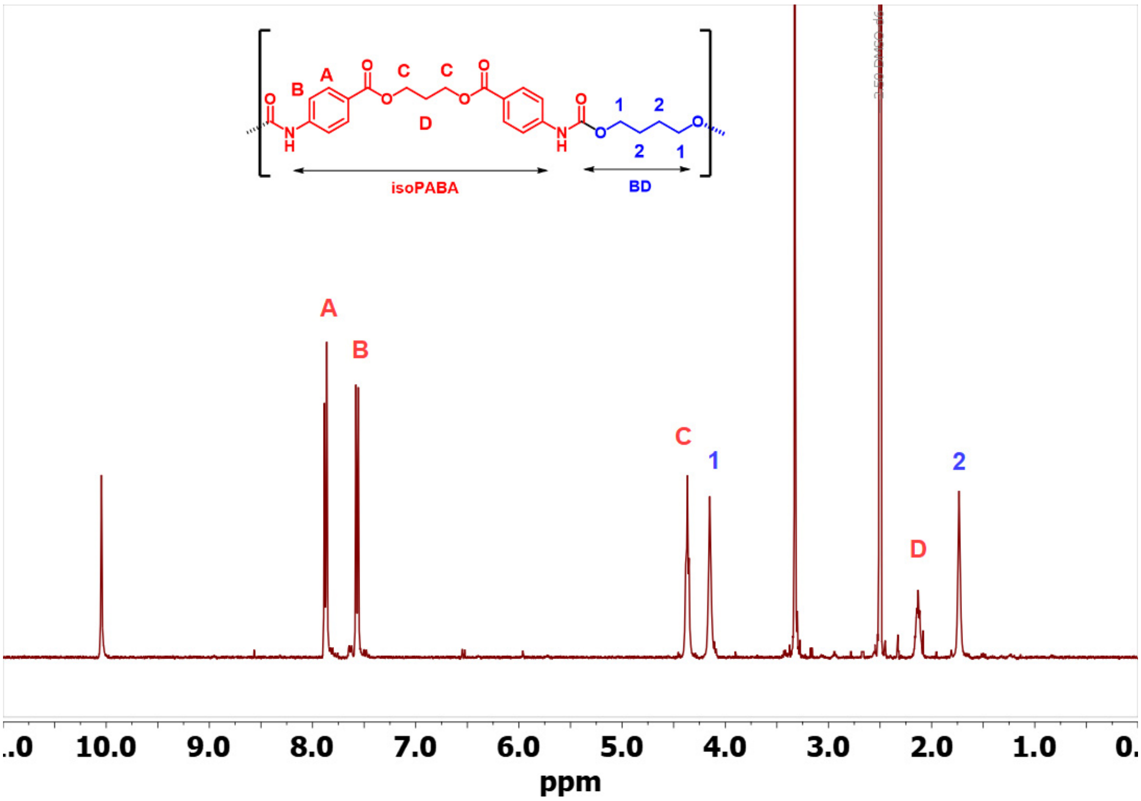

Figure S4: 1H-NMR spectrum of model polyurethane isoPABA-BD in DMSO-d6.

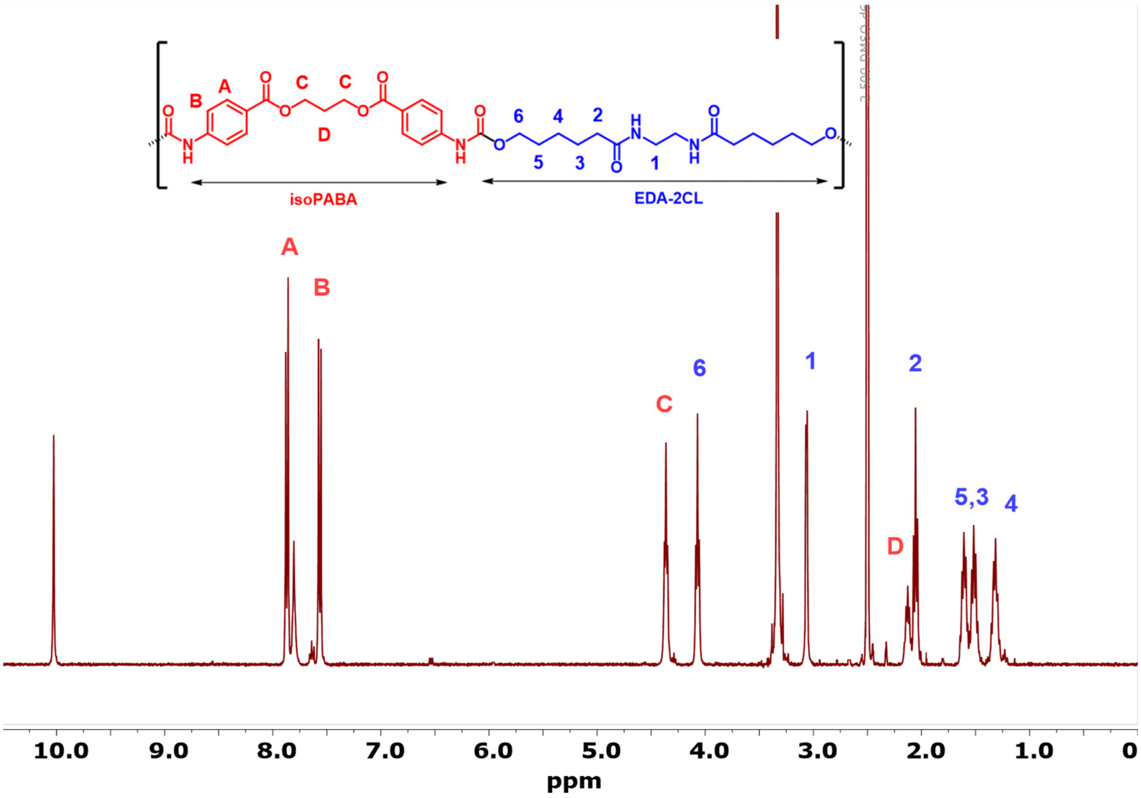

Figure S5: 1H-NMR spectrum of polyurethane isoPABA-EDA-2CL in deuterated DMSO.

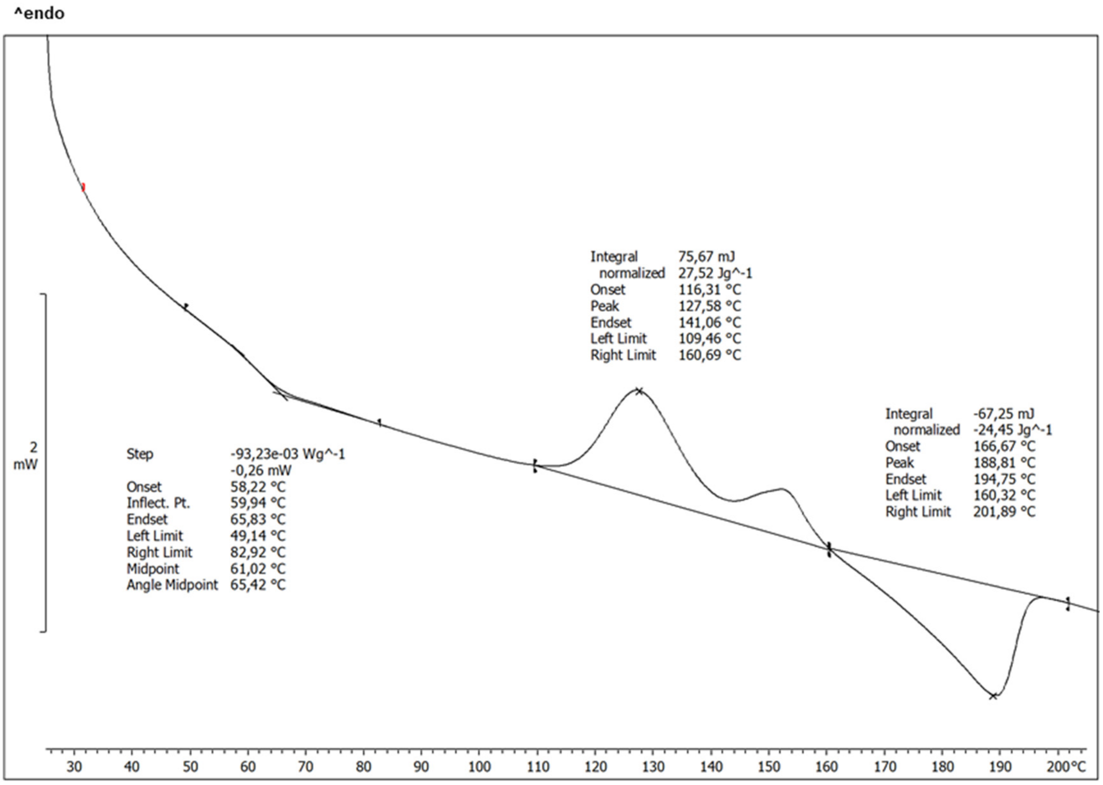

Figure S6: DSC curve of model polyurethane isoPABA-BD.

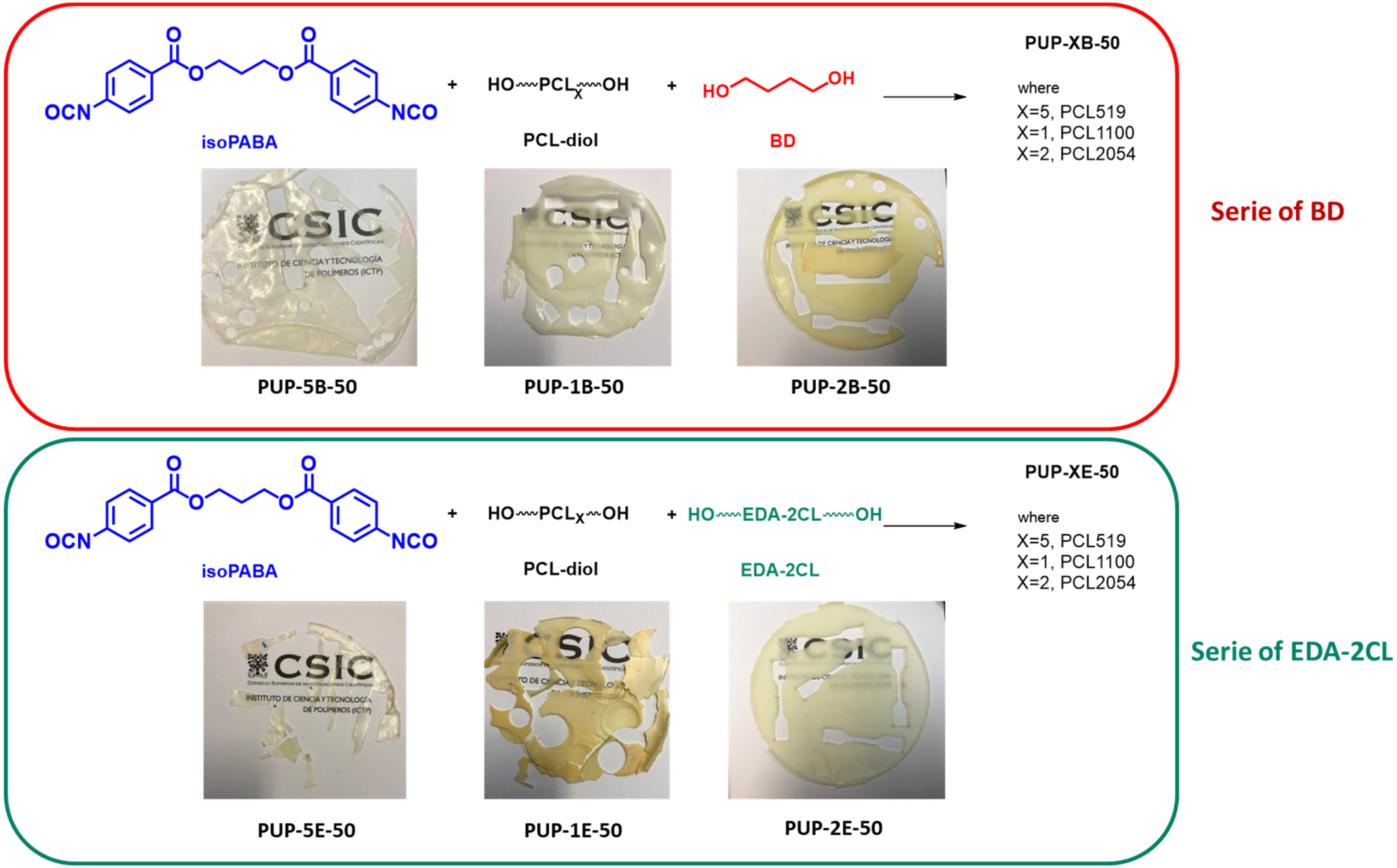

Figure S7: Synthesis scheme and images of poly(ester-urethane)s.

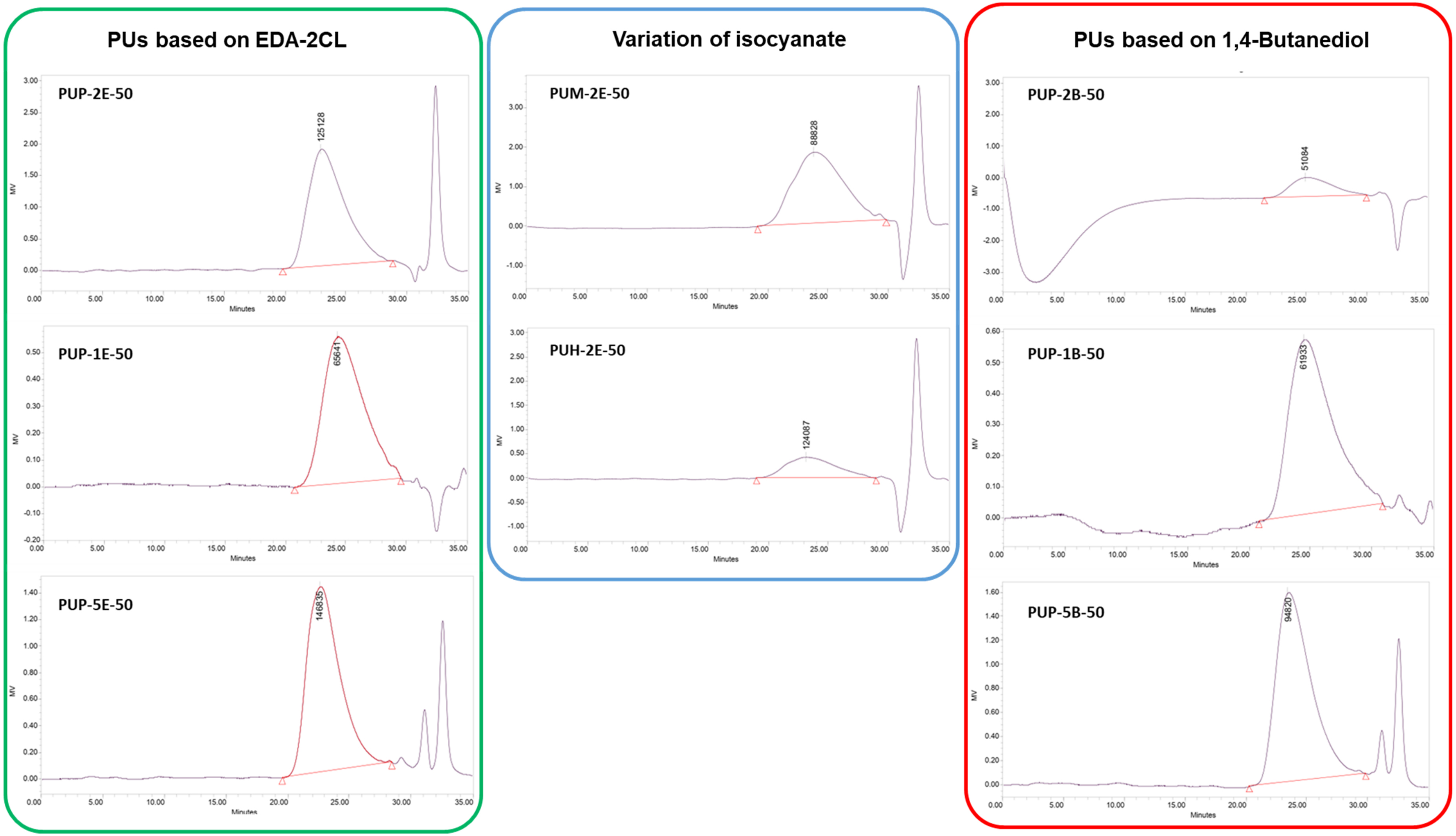

Figure S8. GPC traces of synthesised poly(ester-urethane)s.

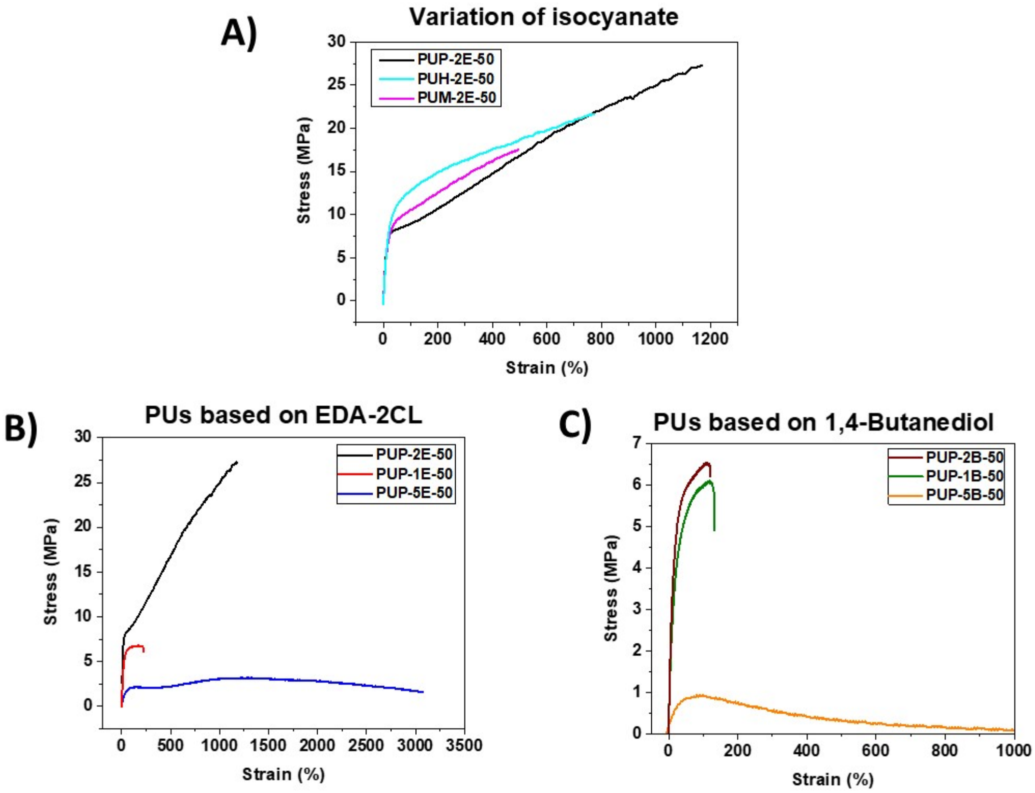

Figure S9: Stress-strain curves of synthetised poly(ester-urethane)s.

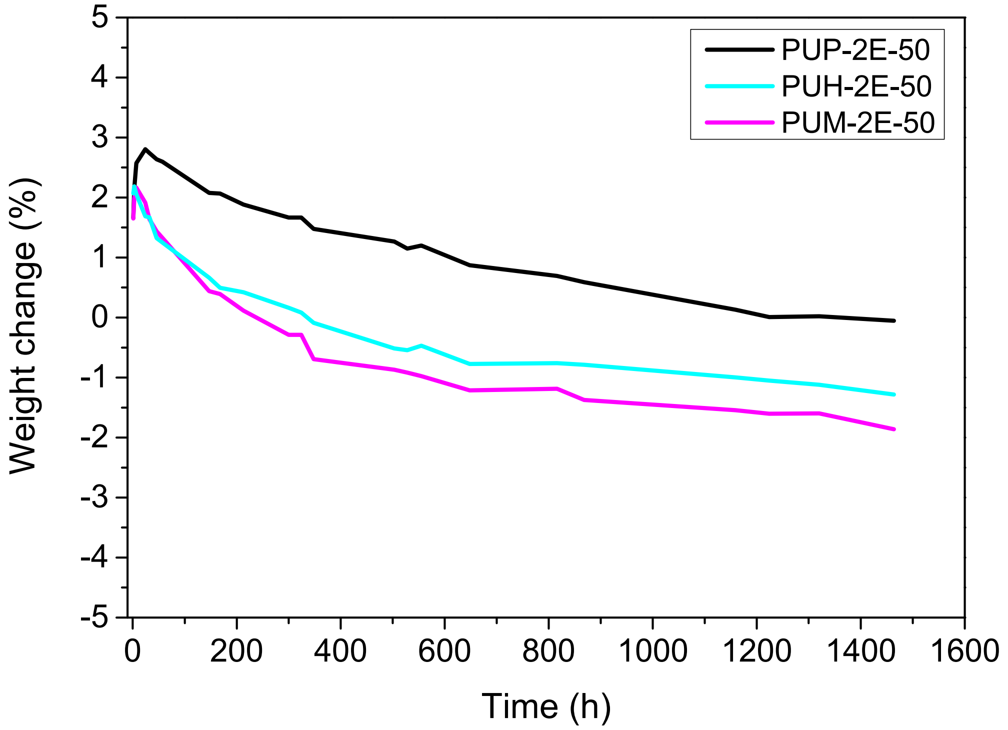

Figure S10: Weight change with immersion time in phosphate buffer solution at 37ºC for hydrated poly(ester-urethane)s based on PCL2054.

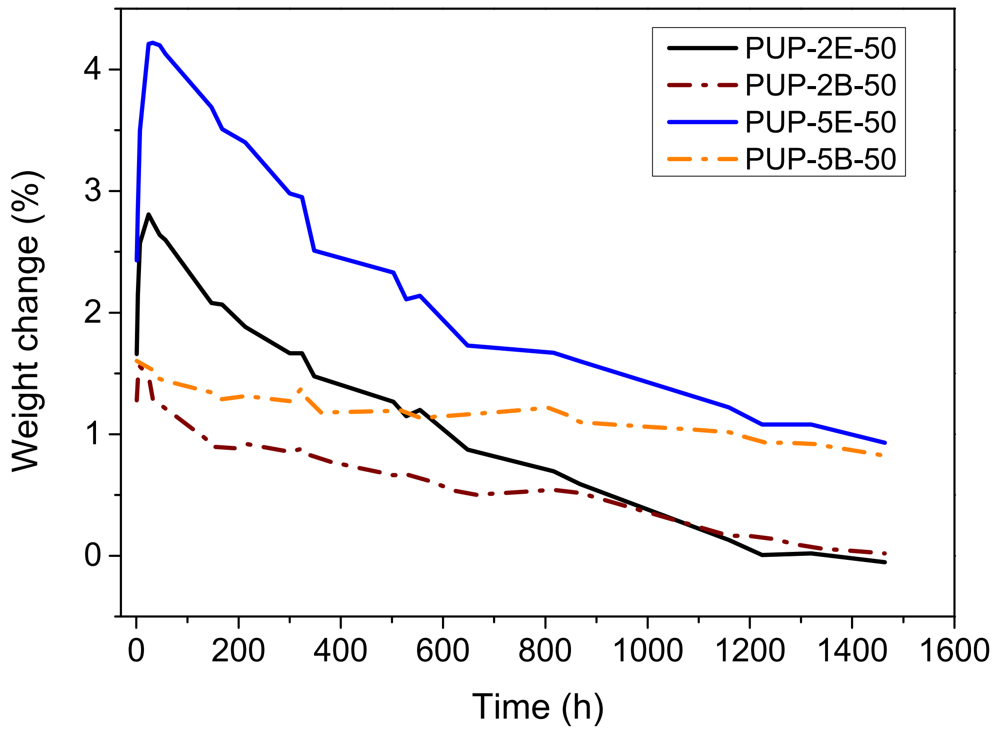

Figure S11: Weight change with immersion time in phosphate buffer solution at 37ºC for hydrated poly(ester-urethane)s with different chain extender.
